# Structure, Antiferroelectricity and Energy-Storage Performance of Lead Hafnate in a Wide Temperature Range

**DOI:** 10.3390/ma16114144

**Published:** 2023-06-02

**Authors:** Vidhi Chauhan, Bi-Xia Wang, Zuo-Guang Ye

**Affiliations:** Department of Chemistry and 4D Labs, Simon Fraser University, Burnaby, BC V5A 1S6, Canada; vidhi_chauhan@sfu.ca (V.C.); bixia_wang@sfu.ca (B.-X.W.)

**Keywords:** lead hafnate, antiferroelectrics, intermediate phase, energy storage

## Abstract

Lead hafnate (PbHfO_3_) has attracted a lot of renewed interest due to its potential as antiferroelectric (AFE) material for energy storage. However, its room temperature (RT) energy-storage performance has not been well established and no reports on the energy-storage feature of its high-temperature intermediate phase (IM) are available. In this work, high-quality PbHfO_3_ ceramics were prepared via the solid-state synthesis route. Based on high-temperature X-ray diffraction data, the IM of PbHfO_3_ was found to be orthorhombic, Imma space group, with antiparallel alignment of Pb^2+^ ions along the [001]_cubic_ directions. The polarization–electric field (P–E) relation of PbHfO_3_ is displayed at RT as well as in the temperature range of the IM. A typical AFE loop revealed an optimal recoverable energy-storage density (*W_rec_*) of 2.7 J/cm^3^, which is 286% higher than the reported data with an efficiency (η) of 65% at 235 kV/cm at RT. A relatively high *W_rec_* value of 0.7 J/cm^3^ was found at 190 °C with an η of 89% at 65 kV/cm. These results demonstrate that PbHfO_3_ is a prototypical AFE from RT up to 200 °C, making it a suitable material for energy-storage applications in a wide temperature range.

## 1. Introduction

While ferroelectric (FE) materials with a parallel arrangement of dipoles in the polar directions have found a wide range of applications such as in transduction sensors, actuators, non-volatile memory, photovoltaics, etc., [1,2,3] antiferroelectric (AFE) materials with an antiparallel alignment of adjacent dipoles are promising candidates for energy-storage applications due to their high-power density, good reliability, long working lifetime, high charge-discharge rate and excellent thermal stability [4,5,6,7]. The energy-storage capability of AFE arises from the electric-field-induced phase transition from the AFE state into an FE state, which is associated with the switching of antiparallel dipoles to parallel dipoles. Despite their great potential, antiferroelectric materials generally suffer from some serious drawbacks such as limited energy density, low dielectric breakdown strength (DBS) and high critical electric fields (E*_cr_*) needed to induce the AFE–FE transition [5,8]. To overcome these issues many lead-based and lead-free solid solutions have been studied in the past few decades. Among them, the most widely explored AFE material is lead zirconate (PbZrO_3_). 

Being a prototypical antiferroelectric material, PbZrO_3_ has been studied in terms of structure, phase transitions and electrical properties. It adopts an orthorhombic structure of Pbam space group at room temperature, with the octahedral tilting following the a^−^a^−^c^0^ scheme (according to the Glazer notation) [9]. On the other hand, lead hafnate (PbHfO_3_), which is isostructural to PbZrO_3_, has not been studied thoroughly. The room temperature symmetry of PbHfO_3_ was reported to be Pbam, which is analogous to PbZrO_3_. However, the structure and symmetry of the intermediate phase and its AFE properties remain under debate. Based on the X-ray diffraction pattern, Kupriyanov et al. proposed that the intermediate phase is polar, but is composed of a mixture of two symmetries, C2mm and P4mm [10]. On the other hand, Bosak et al. suggested a centrosymmetric orthorhombic symmetry with the Imma(00γ)s00 super space group which is linked to incommensurate modulations mainly due to the ordered lead displacements [11]. 

For PbZrO_3_, it is difficult to realize the electric-field-induced AFE-to-FE phase transition at room temperature due to its extremely high E*_cr_* which is greater than its DBS. Thus, substitutions of cations such as La and Sn in PbZrO_3_ were carried out to help not only in decreasing its E*_cr_* and increasing its DBS, but also in realizing AFE double hysteresis loops, so as to increase its recoverable energy-storage density (*W_rec_*), which reached 10.4 J/cm^3^ when the solid solution was prepared via the rolling process [11,12,13]. However, for PbHfO_3_, no clear evidence for room-temperature and high-temperature double P–E loops and thus no energy-storage performance has been reported for conventionally prepared stoichiometric PbHfO_3_. Earlier, similar to PbZrO_3_, it was difficult to detect the electric-field-induced AFE-to-FE transition in PbHfO_3_ since its E*_cr_* is larger than its DBS [14]. Later, the substitutions of various elements into the lattice of PbHfO_3_ led to the observation of double hysteresis loops upon reducing the material’s E*_cr_*. Table 1 compares the key properties including recoverable energy density (*W_rec_*), energy-storage efficiency (η) and dielectric breakdown strength (DBS) of lead hafnium and its solid solutions, along with their respective methods of preparation.

By analyzing Table 1 above, it is apparent that the *W_rec_* of PbHfO_3_ varies depending on the method of preparation used. Gao et al. were not able to display the AFE double hysteresis loop for PbHfO_3_ at room temperature even at an applied electric field of around 80 kV/cm [8]. Wei et al. only obtained a very slim AFE loop with a weak *W_rec_* of 0.7 J/cm^3^ at room temperature for PbHfO_3_ prepared via solid-state reaction. Nevertheless, a much higher *W_rec_*, 7.6 J/cm^3^, was found for the ceramic prepared via the rolling process [21]. Recently, Ge et al. obtained a *W_rec_* of 7.9 J/cm^3^ via the conventional solid-state method but they added an excess amount of PbO powder during the sample preparation [20]. The abovementioned studies present inconsistent values of *W_rec_* at room temperature for PbHfO_3_, especially when the ceramic samples were prepared via the conventional solid-state reaction. However, due to the high volatilization of lead oxide at high temperatures, it is challenging to prepare high-density and pure-phase PbHfO_3_ experimentally. Moreover, the addition of excess PbO in the preparation could raise the question of maintaining stoichiometry and the concern over lead contamination. It is thus of foremost importance to assess the room temperature energy-storage performance of PbHfO_3_ stoichiometrically prepared via solid-state route, so as to design better PbHfO_3_-based materials with high *W_rec_* for energy-storage applications.

On the other hand, as mentioned above, it was suggested that the intermediate phase of PbHfO_3_ is linked to an incommensurate structure; however, Holder et al. showed a nearly ferroelectric-like hysteresis loop for PbHfO_3_ single crystals in the temperature range between 197 °C and 204 °C of the intermediate phase [22]. These contradictory results raise the following questions: (i) If the intermediate phase were a centrosymmetric orthorhombic structure with the Imma(00γ)s00 super space group, how could a nearly ferroelectric-like P–E loop be displayed? (ii) What is the true nature of the intermediate phase? (iii) How are the Pb^2+^ ions arranged in the intermediate phase? 

To answer these questions, the structure and properties of the prototypical AFE PbHfO_3_ are investigated systematically in this work. Highly dense stoichiometric PbHfO_3_ ceramics were prepared via the solid-state synthesis and sintering route. The crystal structure of the intermediate phase was refined and proved to be AFE with the Imma space group. The microstructure, energy-storage density, electric breakdown strength and temperature-dependent dielectric properties were studied. The results reveal that PbHfO_3_ exhibits AFE behavior with double hysteresis loops in both the room-temperature phase and in the intermediate phase with a high recoverable energy density, making pure PbHfO_3_ a viable material for energy-storage applications in a wide range of temperatures. 

## 2. Materials and Methods

### 2.1. Experimental Procedure

In this work, PbHfO_3_ was prepared via the solid-state reaction method. Reagent-grade oxides of PbO (99.9%, Alfa Aesar, Ward Hill, MA, USA) and HfO_2_ (98%, Aldrich Chemistry, Milwaukee, WI, USA) were used as starting materials. All the raw materials were weighed and mixed according to their stoichiometric ratios. The mixed powders were hand-ground in the presence of ethanol for two hours. The slurry was dried at room temperature and pressed into pellets of 20 mm diameter under a pressure of 200 MPa. To prevent the volatilization of PbO at high temperatures, calcination was performed at 850 °C for 4 h via a double-crucible method [23]. In this method the pellets were placed on an alumina plate which was covered with a small Al_2_O_3_ crucible (the diameter of the crucible was large enough to cover all the pellets). Extra PbO powder was added on top of the small alumina crucible. The small alumina crucible with the pellets inside was then covered with another alumina crucible with a larger diameter. The extra PbO helped to provide a high PbO partial pressure at high temperatures and, thereby, inhibited the volatilization of PbO from the pellets. The calcined pellets were then crushed into powder using hand-grinding for 2 h. The dried powder was mixed with 5 wt% PVA as binder and pressed into pellets with a diameter of 10 mm and a thickness of about 1 mm. To obtain high-density ceramics, the pellets were sintered at 1100 °C for 4 h. During the sintering process, the pellets were submerged into sacrificial powder of the same composition so that the volatilization of PbO at high temperatures was further minimized. The sintered ceramics were pale white in color. To study the electrical properties, the ceramics were polished on the circular faces which were then covered with silver paste as electrodes. The samples were fired at 550 °C for 30 min to provide good ohmic contact. 

The crystal structure, phase purity and lattice parameters were determined using X-ray powder diffraction (high resolution Bruker (Billerica, MA, USA), D8 Advance diffractometer with a copper Kα1 X-ray tube) on the fine powder obtained by crushing the as-sintered pellets. Helium ion beam microscopy was used to examine the surface morphology and microstructure of the as-sintered pellets (Zeiss ORION NanoFab, Jena, Germany). An acceleration voltage of 25 kV and an aperture of 10 μm were used to obtain an ion current of 0.153 pA. As the samples were non-conductive in nature, the sample was tilted to an angle of 0.14° to obtain clear and good quality images, and the charge compensation was achieved using the flood gun, together with a line averaging over 16 lines and a dwell time of 500 μs.

The dielectric permittivity was measured from room temperature to 300 °C using a Novocontrol Alpha high-resolution broadband dielectric spectrometer equipped with a temperature-controlled Novotherm HT furnace over a frequency range of 100 Hz to 1 MHz. The samples used to measure the dielectric properties had a thickness of approximately 0.8 mm. Polarization vs. electric field (P–E) hysteresis loops were displayed at 10 Hz on ceramic samples of a thickness of around 0.15 mm using a standard ferroelectric analyzer (Radiant RT66A Standard Ferroelectric Testing System (Radiant Technologies inc., Albuquerque, NM)) at room temperature, and the loops at high temperatures were measured using a DELTA 9023 furnace (Delta Design Inc., CA, USA).

### 2.2. Principles of Energy-Storage Capacitors

A capacitor typically consists of two electrically conductive plates separated by a dielectric layer. When the capacitor is charged, electrical energy is stored within the dielectric. The ability to store energy in a dielectric capacitor is dependent upon its capacitance (C) which can be expressed as [24,25]

(1) $$C=\varepsilon_{r}\;\varepsilon_{0}\frac{A}{d},$$

where ε_r_ is the relative permittivity, ε_0_ is the permittivity of the vacuum (≈8.85 × 10^−12^ F m^−1^) and *A* and *d* are the area of the conducting plate and the distance between the two parallel plates, respectively. The charging process begins as soon as the dielectric capacitor is placed under an applied electric field, and it terminates when the potential generated by the accumulated charges (±Q) on the opposite plates is equal to the applied voltage (*v*). The capacitance, C, can be defined as the incremental change in charge with respect to applied voltage as

(2) $$C=\frac{dq}{dv},$$

and the amount of electrostatic energy stored can be calculated as

(3) $$W=\int_{0}^{Q_{max}}vdq$$

Generally, the density of charges on the surface of the plates is equal to the electrical displacement (=ε_r_ε_0_*E*) in the dielectric material. Thus, the expression for energy stored per unit volume (stored energy density *W_st_*) can be given as

(4) $$W_{st}=\frac{\int_{0}^{Q_{max}}vdq}{Ad},$$

which can be further rewritten as

(5) $$W_{st}=\int_{0}^{D_{max}}EdD,$$

where the electric field *E* is equal to *v*/*d* and *D_max_* is the electric displacement under the maximum applied electric field *E_max_*. 

For ferroelectric materials *D* = Polarization, *P*, so Equation (5) can be written as

(6) $$W_{st}=\int_{0}^{P_{max}}EdP,$$

or as

(7) $$W_{st}=\int_{0}^{P_{max}}EdP\;\;\;\;\;\;\;\;\;\;\left(0<E<E_{max}\right),$$

and the recoverable energy density is

(8) $$W_{rec}=-\int_{P_{max}}^{P_{r}}EdP<W_{st},$$

where *P_max_* and *P_r_* are the maximum polarization and remanent polarization, respectively.

## 3. Results and Analysis

### 3.1. Structural Analysis

**Figure 1:**
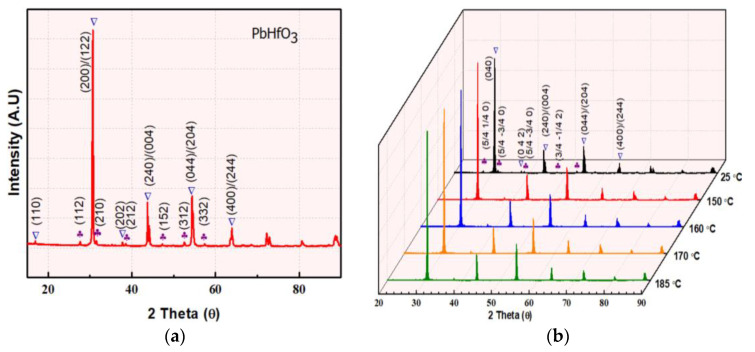

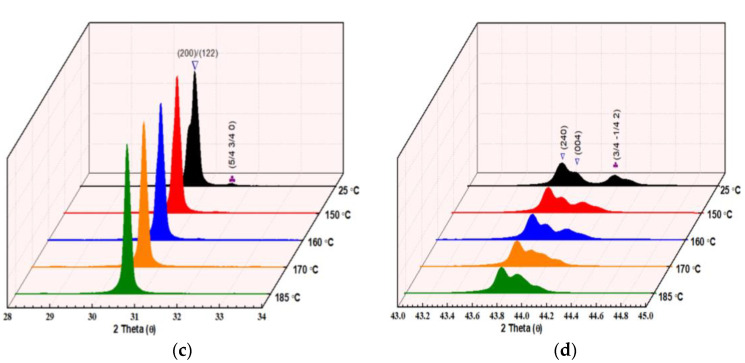
(**a**) XRD patterns of lead hafnate PbHfO_3_ measured at room temperature, with the peaks labeled in the orthorhombic setting. (**b**) XRD patterns of PbHfO_3_ measured at different temperatures. (**c**,**d**) Enlarged views of the ¼ (hkl) super lattice peaks at 25 °C, 150 °C, 160 °C, 170 °C and 185 °C.

The room temperature powder XRD pattern of an as-sintered PbHfO_3_ ceramic is presented in Figure 1a. Refinement of all the major peaks (marked by the ∇ symbol) with the help of GSAS2full 4801 software [26] revealed that the compound crystalizes into a perovskite structure with an orthorhombic symmetry of the Pbam space group. No pyrochlore phase was observed. The visible low-intensity peaks identified by “♣” represent the ¼ (hkl) superlattice peaks. These superlattice peaks are characteristic of the antiferroelectric structure of PbHfO_3,_ as they appear due to the antiparallel displacements of Pb^2+^ ions along the pseudocubic [110]_pc_ directions [10,27,28]. To better represent the superlattice peaks and to study the structural change more closely, the orthorhombic lattice setting of √2a_pc_ × 2√2a_pc_ × 2a_pc_ was converted into a pseudocubic lattice setting, a_pc_ × a_pc_ × a_pc_, by using the following transformation matrix [27]:

(9) $$(h_{0}\;k_{0}\;l_{0})\left(\begin{array}{ccc} \frac{1}{2} & -\frac{1}{2} & 0\\ \frac{1}{4} & \frac{1}{4} & 0\\ 0 & 0 & \frac{1}{2} \end{array}\right)=(h_{\mathrm{pc}}k_{\mathrm{pc}}l_{\mathrm{pc}}),$$

where a_pc_ is the cell dimension of the para-electric cubic phase.

To investigate the symmetry of the intermediate phase, temperature-variable structural analysis was performed. Figure 1b depicts the powder XRD patterns of PbHfO_3_ measured at selected temperatures between room temperature and 185 °C (powder XRD at temperatures higher than 200 °C was not carried out as it is beyond the limit of our instrument). Distinctly, the (200)/(122) peaks merge in to a single peak (Figure 1c) at 160 °C, and, at the same time, the shape of the (240)/(004) peaks (Figure 1d) changes significantly. These indicate a structural change from the orthorhombic Pbam symmetry to an orthorhombic Imma symmetry (to be discussed later). Moreover, the diminishing intensity of the superlattice peaks with increasing temperature also supports the change in symmetry. 

**Figure 2:**
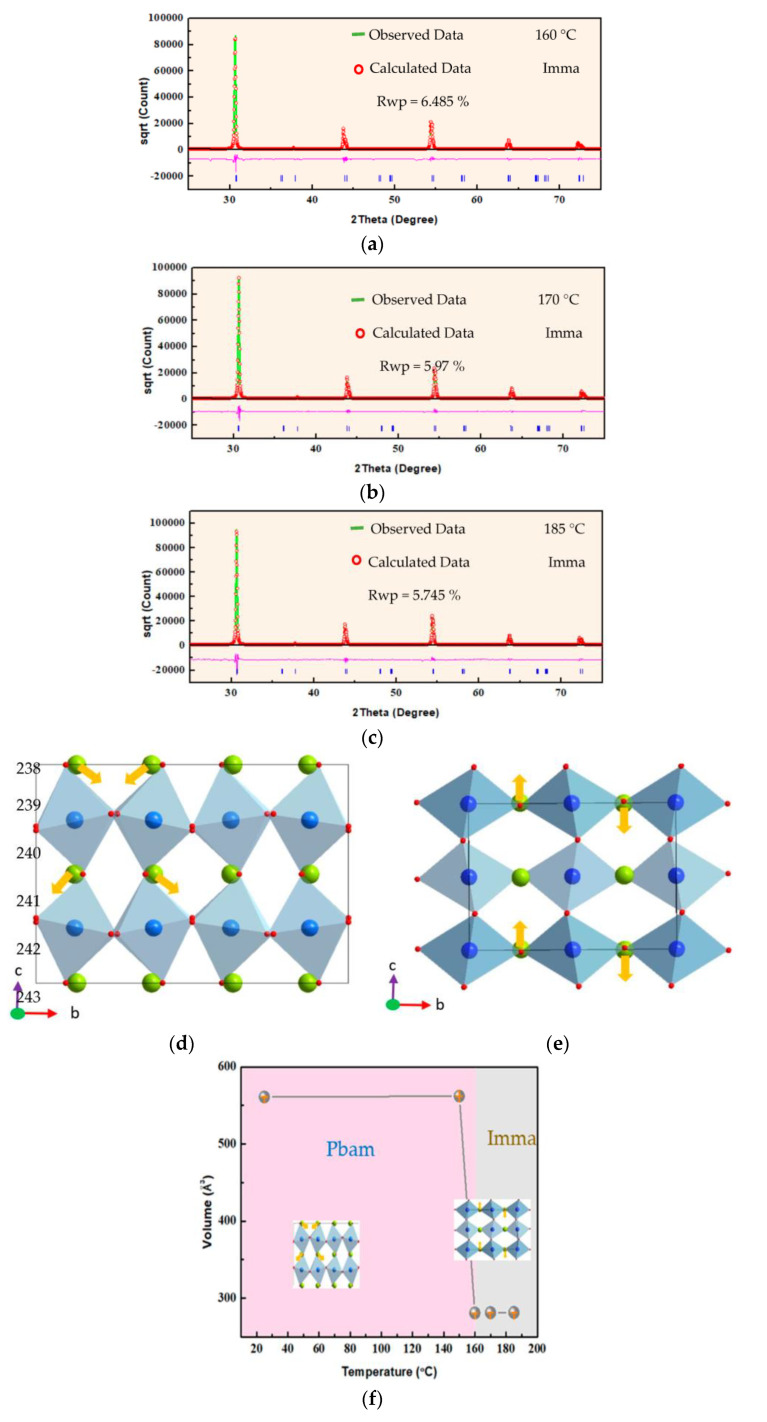
(**a**–**c**) Rietveld refinement results of PbHfO_3_ at 160 °C, 170 °C and 185 °C. (**d**) Refined crystal structure of PbHfO_3_ at room temperature with the Pbam space group, viewed along the a-axis. (**e**) Refined crystal structure of PbHfO_3_ at 185 °C with the Imma space group, viewed along the a-axis. Light blue polyhedrons stand for the HfO_6_ octahedrons. Blue and green spheres denote the Pb and Hf atoms, respectively. Yellow arrows stand for the displacements of the Pb^2+^ ions. (**f**) Variation of the unit cell volume of PbHfO_3_ as a function of temperature between the Pbam and Imma phases.

To investigate the abovementioned phase transition, a detailed analysis of the structure of PbHfO_3_ in the intermediate phase was carried out using the Rietveld refinements of the high-temperature X-ray diffraction data using the GSAS2full 4801 software. Figure 2a–e present the refinement results for the intermediate phase, with comparison of the experimental and calculated XRD patterns, along with the respective space groups and reliability factors. Since no superlattice reflections of the 1/4 (hkl)-type were found in the temperature range of the intermediate phase, the Pbam space group can be excluded, while the Imma space group fits the experimental data most satisfactorily. The assignment of the Imma space group to the intermediate phase of PbHfO_3_ agrees with a report by Bosak et al. [11]. The details of the Rietveld refinement results of PbHfO_3_ at different temperatures within the intermediate phase are given in Table 2. The reliability factors of the weighted patterns (R*_wp_*), the final refinement (wR) and the goodness of fit (GOF) are in the ranges of 3.434–6.485%, 4.926–6.49% and 1.513–2.32%, respectively, indicating good fitting of the selected structural model. At high temperatures, no oxygen octahedral tilting about the a-axis was observed, as opposed to at room temperature. The tilting scheme for the Imma phase is a^0^b^-^b^-^, while at room temperature; the Pbam symmetry shows the a^−^a^−^c^0^ tilting scheme, similar to that in lead zirconate [9,28,29,30]. The antiparallel displacement of Pb^2+^ ions was found to be along the [001]_cubic_ direction in the intermediate phase, as shown in Figure 2e, which is clearly different from the alignment of Pb^2+^ ions at room temperature, as shown in Figure 2d. This antiparallel dipolar alignment suggests an AFE nature for the intermediate phase. The change in the lattice volume with temperature is presented in Figure 2f. It can be seen that the unit cell volume was drastically decreased upon heating at about 160 °C, as PbHfO_3_ underwent the transition from the room temperature Pbam phase to the intermediate phase of the Imma space group.

### 3.2. Dielectric Properties

**Figure 3:**
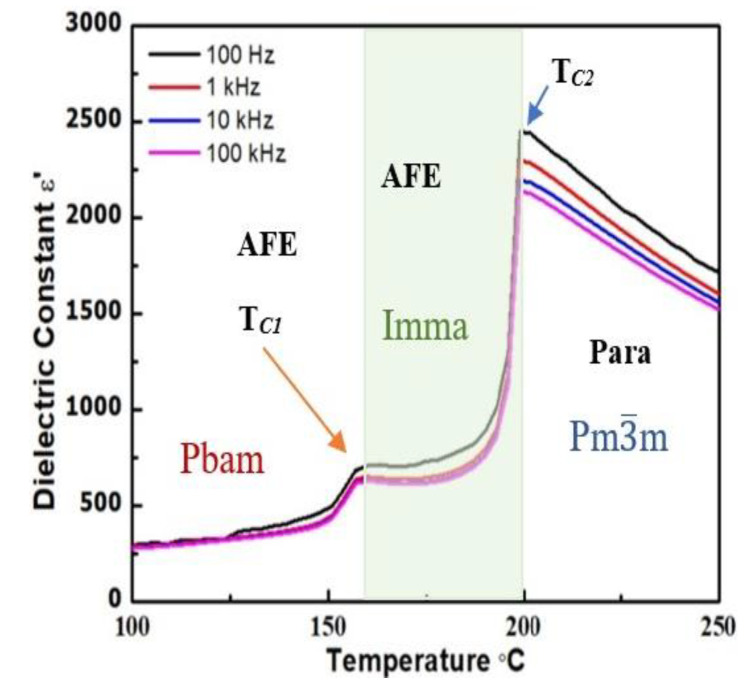
Temperature dependence of the dielectric permittivity of a PbHfO_3_ ceramic measured at various frequencies showing the phase transitions from Pbam to Imma, and then from Imma to Pm$\overset{-}{3}$m, upon heating.

Figure 3 depicts the temperature and frequency dependencies of the dielectric permittivity of an as-sintered ceramic measured at various frequencies upon heating. Two dielectric anomalies can be clearly found at 163 °C (T*_C_*_1_) and 199 °C (T*_C_*_2_), which correspond to the phase transitions from the antiferroelectric orthorhombic Pbam phase to the antiferroelectric orthorhombic Imma phase, and from the antiferroelectric Imma phase to the paraelectric, cubic Pm$\overset{-}{3}$m phase, respectively. 

### 3.3. Microstructure and Energy-Storage Performance

**Figure 4:**
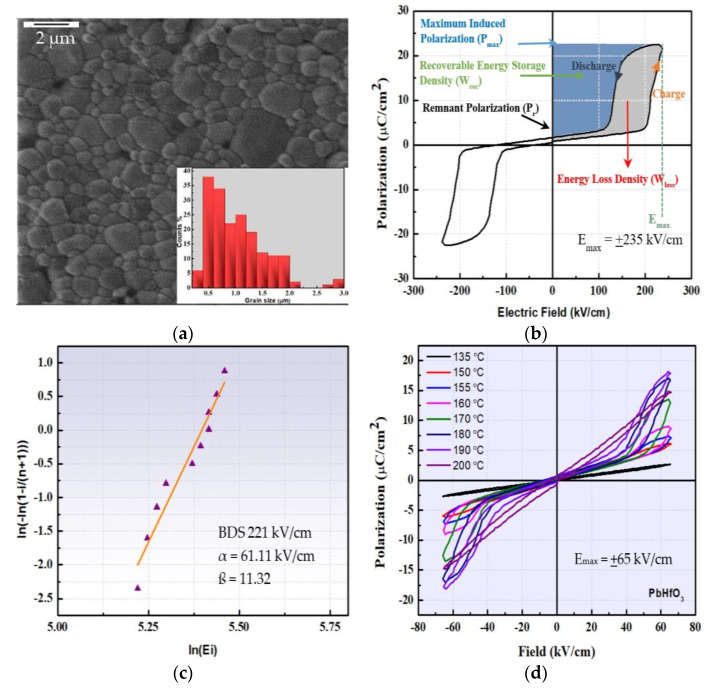
(**a**) HIM image of a PbHfO_3_ ceramic (scale bar = 2 μm) with the average grain size distribution presented in the inset. (**b**) Polarization–electric field (P–E) relation of PbHfO_3_ displayed at room temperature; the blue area constitutes the recoverable energy density (*W_rec_*) and the grey area represents the energy loss (*W_loss_*). (**c**) The Weibull distribution of the PbHfO_3_ ceramics used for the calculation of the DBS. (**d**) The polarization–electric field (P–E) relations measured at an electric field of +65 kV/cm at high temperatures.

To reveal the AFE character of PbHfO_3_, which is primordial for energy-storage applications, it is necessary to obtain high-density ceramics with optimum microstructures, so as to apply a high electric field and to realize the AFE to FE switching. Figure 4a presents the HIM image of a PbHfO_3_ ceramic, which shows a dense microstructure achieved via the conventional sintering process described in Section 2. The average relative density of the ceramics reached 95% of the theoretical density of 10.27 g/cm^3^. The average grain size of the ceramic calculated with the help of ImageJ 1.8.0 software was about 0.5 μm. The graph of the average grain size distribution is presented in the inset of Figure 4a. 

The polarization–electric field (P–E) relation of PbHfO_3_ measured at room temperature at 10 Hz is depicted in Figure 4b. The P–E relation exhibits a typical AFE double hysteresis loop, which results from the electric-field-induced AFE-to-FE phase transition, demonstrating the antiferroelectricity of PbHfO_3_ at room temperature thanks to the high quality of the ceramics. The maximum induced polarization was 23 μC/cm^2^ at an applied electric field of 235 kV/cm and the remanent polarization was 1.63 μC/cm^2^ when the applied electric field returned to zero. Theoretically, the stored energy density (*W_st_*) and recoverable energy density (*W_rec_*) can be calculated as discussed in Section 2.2.

The calculated stored energy density *W_st_*, recoverable energy density *W_rec_*, energy loss *W_loss_* and efficiency ɳ were found to be 4.216 J/cm^3^, 2.7 J/cm^3^, 1.5 J/cm^3^ and 65%, respectively, with the energy-storage efficiency calculated from [31]:

(10) $$ɳ=\frac{W_{rec}}{W_{st}}=\frac{W_{rec}}{W_{rec}+W_{loss}}\times 100\%$$

The dielectric breakdown strength (DBS) is one of the most important factors affecting energy-storage performance. It is highly influenced by the density, grain size and microstructure of the ceramics. Dense ceramics with smaller grain size tend to exhibit higher DBS values. Figure 4c presents the Weibull distribution of the DBS for the PbHfO_3_ ceramics, which can be expressed as [31,32,33,34,35,36]

X_i_ = ln (E_i_), (11)

Y_i_ = ln (ln (1/(1 − P_i_))), (12)

and

P_i_ = i/(n + 1), (13)

where X_i_ and Y_i_ are the parameters in the Weibull distribution function, E_i_ is the specific breakdown electric field of the specimen, P_i_ is the polarizability, n is the sum of specimens and i is the serial number of the specimen. To obtain the average breakdown strength of PbHfO_3_, ten samples were examined and their data taken into consideration. The shape parameter ß is the slope of the line, and is related to the range of the DBS. The intercept on the *X*-axis is ln(α), where α is the scale parameter which reflects the magnitude of the DBS. The values of ß and α were found to be 11.32 and 61.11 kV/cm, respectively. All the dielectric breakdown data were found to follow the Weibull distribution and the obtained DBS value for PbHfO_3_ was 221 kV/cm. The values of ß and the DBS are in good agreement with previously reported data [14]. 

Figure 4d portrays the polarization–electric field (P–E) relation of PbHfO_3_ measured at ±65 kV/cm in the temperature range from 150 to 190 °C. Well-defined double hysteresis loops clearly demonstrate the antiferroelectric nature for the intermediate Imma phase. The display of the AFE loops is in strong support of the antiparallel alignment of Pb^2+^ ions along the [001]_cubic_ direction, as depicted from the refined crystal structure of PbHfO_3_. The highest polarization of 18.0 μC/cm^2^ was obtained at 190 °C with a very small value of remanent polarization of 0.6 μC/cm^2^. The calculated *W_st_*, *W_rec_*, *W_loss_* and ɳ were 0.774 J/cm^3^, 0.7 J/cm^3^, 0.084 J/cm^3^ and 89%, respectively, at 190 °C. 

## 4. Discussion 

Environmental concerns related to lead: Environmental concerns related to the safety of hazardous materials, such as lead oxide, involved in processing, distribution, transportation, and final decomposition are important issues and require careful consideration. In our case, to ensure the safety, the lead hafnate ceramics were sintered and calcined using a muffle furnace which was kept under a high-power ventilation hood. This effectively prevented the volatile materials from escaping into the air, thereby mitigating potential health hazards associated with the exposure of lead oxide during the processing. The double crucible method used in this work for calcining and sintering helped further minimize the volatilization of lead oxide. During transportation, the raw materials were properly packed, contained and labelled [37,38]. For the disposal of lead-based materials, they could be converted into nontoxic products or metals and recycled as solid waste [39,40].

Economics of scale achievement: The solid-state synthesis route developed in this work offers several advantages over other processing routes, such as wet chemical methods, by its simplicity and need for less equipment and is thus a viable technique for scaling up industrial production of lead hafnate for practical applications. Additionally, the raw materials used in the solid-state synthesis are relatively inexpensive, making it a cost-effective option for commercial manufacturing [41].

In an effort to clarify the structural nature and AFE properties of lead hafnate, high-quality and dense PbHfO_3_ ceramics were prepared in pure perovskite phase using the solid-state synthesis method and a conventional sintering process with special caution to preserve the stoichiometry. Detailed structural analysis using the Rietveld refinements confirmed the orthorhombic symmetry with the Pbam space group at room temperature which is in agreement with earlier reports [10,28]. Temperature-variable structural analysis revealed an orthorhombic Imma symmetry for the intermediate phase of lead hafnate between 150 °C and 185 °C, which agrees with a report by Bosak et al. [11]. The refined crystal structure of the Imma phase illustrates antiparallel displacement of Pb^2+^ ions along the [001]_cubic_ direction with the tilting scheme of a^0^b^-^b^-^, which is different from the antiparallel alignment of Pb^2+^ ions in the Pbam phase at room temperature. 

The high quality of the ceramics allowed us to apply a high enough electric field so as to display the P–E double hysteresis loops, demonstrating the AFE nature of PbHfO_3_ at room temperature and also at high temperatures (up to 190 °C). The realization of the double P–E loops also allowed us to evaluate the energy-storage performance of PbHfO_3_. The recoverable energy density was found to be 2.7 J/cm^3^ with an efficiency of 65% at 235 kV/cm at room temperature. According to the data presented in Table 1, the energy-storage performance of the lead hafnate ceramics prepared in this work is 286% higher than the values reported so far for the conventionally prepared stoichiometry PbHfO_3_ without the addition of extra PbO. This enhanced performance is attributed to the high density and small grain size of the ceramics, which lead to a high value of dielectric breakdown strength, DBS, allowing the antiferroelectric-phase-to-ferroelectric-phase transition to be induced at room temperature. 

The double P–E hysteresis loops displayed at high temperatures reveal for the first time the presence of antiferroelectricity in the intermediate phase, with a recoverable energy density of 0.7 J/cm^3^ and an efficiency of 89% at an electric field of 65 kV/cm at 190 °C. 

## 5. Conclusions

To better understand the structural nature and AFE properties of lead hafnate, high-quality and dense PbHfO_3_ ceramics were prepared in pure perovskite phase using the solid-state synthesis method and a conventional sintering process with special caution to preserve the stoichiometry without adding excess PbO. However, due to the high volatilization of lead oxide at high temperatures, it is challenging to prepare high-density and pure-phase PbHfO_3_ experimentally. Rietveld refinements on high-temperature structural analysis revealed an orthorhombic Imma symmetry for the intermediate phase of lead hafnate between 150 °C and 185 °C, with antiparallel displacement of Pb^2+^ ions along the [001]_pc_ direction with the tilting scheme of a^0^b^−^b^−^. This is different from the antiparallel alignment of Pb^2+^ ions in the Pbam phase at room temperature. The realization of the double P–E loops at room temperature also allowed us to evaluate the energy-storage performance of PbHfO_3_. At room temperature, the recoverable energy density was found to be 2.7 J/cm^3^, with an efficiency of 65% at 235 kV/cm at room temperature. The energy-storage performance of lead hafnate prepared in this work is 286% higher than the values reported so far for the conventionally prepared stoichiometry PbHfO_3_. This enhanced performance is attributed to two key factors: the high density and the small grain size of the ceramics. This led to a high value of dielectric breakdown strength, DBS, which allows the transition of antiferroelectric phase to ferroelectric phase at room temperature. The double P–E hysteresis loops displayed at high temperatures reveal, for the first time, the presence of antiferroelectricity in the intermediate phase. These results demonstrate that the prototypical AFE PbHfO_3_ is indeed a promising candidate for energy-storage applications [42] in a wide range of temperatures from room temperature up to 190 °C. 

## Figures and Tables

**Table 1:** Summary of key properties including recoverable energy density (*W_rec_*), energy-storage efficiency (η), dielectric breakdown strength (DBS) and methods of preparation of lead hafnium and lead hafnium-based solid solutions.

Materials	Synthesis Method	*W_rec_* J/cm^3^	η %	DBS kV/cm	Refs.	Year
Pb(Hf_0.98_Ti_0.02_)O_3_	Solid-state reaction with 3 wt% PbO excess	4.15	65.3	190	[15]	2023
(Pb_1−3x/2_La_x_)Hf_0.96_Ti_0.04_O_3_	Solid-state reaction with 3 wt% PbO excess	11.2	88.9	360	[16]	2022
Pb_0.925_Ba_0.045_La_0.03_(Hf_0.6_Sn_0.4_)_0.9925_O_3_	Rolling process	7.3	91	290	[17]	2021
Pb_0.98_La_0.02_(Hf_x_Sn_1−x_)_0.995_O_3_	Rolling process	7.63	94	380	[18]	2020
PbHf_1−x_Sn_x_O_3_	Solid-state reaction with 3 wt% PbO excess	10.2	78.9	320	[19,20]	2020, 2022
(1−x)PbHfO_3_−xPb(Mg_1/2_W_1/2_)O_3_	Two-step solid-state reaction	3.7	72.5	155	[8]	2019
PbHfO_3_	Rolling process	7.6	80.8	270	[21]	2019
PbHfO_3_	Solid-state reaction with 3 wt% PbO excess	7.9	75.3	258	[20]	2022
PbHfO_3_	Solid-state reaction	0.7	-	220	[21]	2019

**Table 2:** Refined structural parameters of PbHfO_3_ at different temperatures.

Temperature °C	Space Group	Ions	x	y	z	R*_wp_* %	wR %	GOF
25	Pbam	Pb^2+^1	0.6985	0.12986	0	3.469	5.106	1.514
		Pb^2+^2	0.7146	0.1238	0.5			
		Hf	0.24284	0.1237	0.2527			
		O1	0.297	0.095	0			
		O2	0.272	0.150	0.5			
		O3	0.033	0.260	0.224			
		O4	0.0	0.5	0.300			
		O5	0	0	0.300			
150	Pbam	Pb^2+^1	0.7137	0.1297	0	3.434	4.926	1.513
		Pb^2+^2	0.7192	0.1247	0.5			
		Hf	0.2446	0.1249	0.2487			
		O1	0.318	0.112	0			
		O2	0.271	0.157	0.5			
		O3	0.02	0.259	0.217			
		O4	0.0	0.5	0.273			
		O5	0	0	0.254			
160	Imma	Pb^2+^1	0.03348	0.75000	0.50355	6.485	6.49	2.32
		Hf	0.0000	0.000	0.000			
		O1	0.0000	0.75000	0.02158			
		O2	0.25000	0.00564	0.25000			
170	Imma	Pb^2+^1	0.02492	0.75000	0.50307	5.97	5.97	2.13
		Hf	0.0000	0.0000	1.0000			
		O1	0.0000	0.75000	0.02432			
		O2	0.2500	0.02760	0.2500			
185	Imma	Pb^2+^1	0.02909	0.75000	0.50520	5.745	5.75	2.05
		Hf	0.0000	0.0000	0.0000			
		O1	0.0000	0.75000	0.01175			
		O2	0.2500	0.02738	0.25000			

## Data Availability

No new data were created or analyzed in this study. Data sharing is not applicable to this article.
